# Infectious bursal disease virus infection leads to changes in the gut associated-lymphoid tissue and the microbiota composition

**DOI:** 10.1371/journal.pone.0192066

**Published:** 2018-02-01

**Authors:** Li Li, Tereza Kubasová, Ivan Rychlik, Frederic J. Hoerr, Silke Rautenschlein

**Affiliations:** 1 University of Veterinary Medicine Hannover, Clinic for Poultry, Bünteweg, Hannover, Germany; 2 Veterinary Research Institute, Hudcova, Brno, Czech Republic; 3 Veterinary Diagnostic Pathology, Fort Valley, Virginia, United States of America; CEA, FRANCE

## Abstract

Infectious bursal disease (IBD) is an acute, highly contagious and immunosuppressive poultry disease. IBD virus (IBDV) is the causative agent, which may lead to high morbidity and mortality rates in susceptible birds. IBDV-pathogenesis studies have focused mainly on primary lymphoid organs. It is not known if IBDV infection may modify the development of the gut associated lymphoid tissues (GALT) as well as the microbiota composition. The aim of the present study was to investigate the effects of IBDV-infection on the bursa of Fabricius (BF), caecal tonsils (CT) and caecum, and to determine the effects on the gut microbiota composition in the caecum. Commercial broiler chickens were inoculated with a very virulent (vv) strain of IBDV at 14 (Experiment 2) or 15 (Experiment 1) days post hatch (dph). Virus replication, lesion development, immune parameters including numbers of T and B lymphocytes, macrophages, as well as the gut microbiota composition were compared between groups. Rapid IBDV-replication was detected in the BF, CT and caecum. It was accompanied by histological lesions including an infiltration of heterophils. In addition a significant reduction in the total mucosal thickness of the caecum was observed in vvIBDV-infected birds compared to virus-free controls (*P* < 0.05). vvIBDV infection also led to an increase in T lymphocyte numbers and macrophages, as well as a decrease in the number of B lymphocytes in the lamina propria of the caecum, and in the caecal tonsils. Illumina sequencing analysis indicated that vvIBDV infection also induced changes in the abundance of *Clostridium XIVa* and *Faecalibacterium* over time. Overall, our results suggested that vvIBDV infection had a significant impact on the GALT and led to a modulation of gut microbiota composition, which may lead to a higher susceptibility of affected birds for pathogens invading through the gut.

## Introduction

Infectious bursal disease virus (IBDV) is the causative agent of infectious bursal disease (IBD) [1]. To date, this disease is prevalent in most of the poultry-producing regions of the world [2, 3]. Very virulent (vv) IBDV infection generally results in immunosuppression, which may lead to high mortality rates in susceptible chickens. vvIBDV-induced immunosuppression in the early phase of the chicken’s growing period may result in subsequent problems with secondary infections including also gut-associated diseases, which contribute to the economic losses in the poultry industry [4].

IBDV targets IgM+ B cells leading to a severe damage of the bursa of Fabricius (BF). In recent years, vvIBDV-pathogenesis studies have mainly focused on the primary lymphoid tissues such as the BF and the thymus. However, only little is known about the effects of vvIBDV on the gut-associated lymphoid tissues (GALT) besides the BF. These comprise organized lymphoid tissues such as caecal tonsils (CT), peyer’s patches (PP), Meckel’s diverticulum and other lymphoid aggregates located within the lamina propria (LP) along the gastrointestinal tract [5]. These establish a first line of defense against invading pathogens and also contribute to systemic immune responses [6]. Previous studies demonstrated that lymphocytes and macrophages in the intestine play a role in vvIBDV transmission to the BF and other sites [7, 8]. vvIBDV may impair the intestinal mucosal immunity. One recent study demonstrated that vvIBDV infection led to a decrease in villus height, and a reduction in the number of intestinal intraepithelial lymphocytes (IEL) and mast cells in the intestine of specific-pathogen-free (SPF) chickens [9]. These effects on gut associated immunity were observed during the first three days after vvIBDV infection.

Little is known about the interaction between vvIBDV and the gut microbiota. The interaction between viruses and the microbiota is presently an area of intensive research in human and other animal models [10]. It has been shown that the immune system is also likely to be an important contributor to host control over microbiota composition [11]. Several cell types such as goblet cells, IgA secreting B cells as well as intraepithelial lymphocytes (IEL) function together to stratify luminal microbes and to minimize bacterial-epithelial contact [12–14]. Likewise it has been shown that microbiota shapes immunity. Studies comparing germ-free and microbiota colonized mice revealed an effect of microbial colonization on the formation of lymphoid tissues and subsequent immune system development [11]. We hypothesize that vvIBDV may lead to a modification of the GALT and subsequently the gut microbiota composition, which enhances the risk of pathogen invasion of the host through the gut [15, 16]. Previously another important immunosuppressive virus of chickens, Marek’s disease virus (MDV), was shown to modify the core gut microbiota composition [17].

Our objective was to investigate the effect of vvIBDV on the GALT of commercial broiler chickens and the gut microbiota composition. In two experiments, broiler chickens were experimentally inoculated with vvIBDV at 14 or 15 day post hatch, when the maternally derived IBDV antibodies (MDA) were confirmed to be below the break through level of the virus. Lesion development, viral antigen load, and local immune cell populations were investigated in selected GALT such as the BF, CT and caecum. In addition, caecum harbors a more diverse microbial community compared to other intestinal sections, and it is physically associated with the CT, therefore caecal content was selected to determine the gut microbiota by 16S rRNA sequencing. Our study clearly demonstrates that vvIBDV not only modified immune cell populations in the BF but also in CT and caecum, and subsequently led to changes in the microbiota composition. This indicates that not only humoral immunity and innate immune parameters are affected in IBDV-infected birds, but also the intestinal barrier is significantly altered, which could allow secondary pathogens to colonize.

## Material and methods

### Animals

One-day-old commercial broiler chicks (Ross 308, mixed sex) were purchased from the hatchery Brüterei Weser-Ems, GmbH & Co. KG, Visbek, Rechterfeld, Germany. All chicks were kept in the same room at the Clinic for Poultry under isolation conditions on wood shavings until the day of inoculation. On this day, the chicks were randomly distributed to different isolation units and subsequently inoculated with virus or phosphate-buffered saline (PBS). All groups received the same feed and water from the same commercial source *ad libitum*. The chicks did not receive any vaccination. Experiments were conducted following the regulations for animal welfare of Lower Saxony and were approved by the Lower Saxony State Office for Customer Protection and Food Safety (LAVES: 33.12-42505-04-13/1215). Each bird was individually marked with a wing tag.

All groups were evaluated daily for clinical signs. The following parameters were determined to evaluate the health status of the animals and clinical scores/animal in case of illness. If clinical disease would have been observed in one group, individual birds with clinical signs such as huddling, ruffled feathers, separation from the group, loss of feathers and skin integrity, dirty feathers, bleeding, nasal or conjunctival discharge would have been identified. Individual birds were more closely investigated then to determine the clinical score. The clinical score is based on the following criteria: breathing and excrement quality, injuries, conjunctiva condition, modification at the blood sampling region, feed and water intake, and locomotion. Each parameter was evaluated and scored from 0 to three ranging from no signs/normal (score 0) to severe signs (score 3) (S1 Table). The maximum total clinical score for all six parameters is 18. The following criteria led to the definition of the humane endpoint: one bird shows at three subsequent controlling time points (at least two observation time points per day) a total clinical score of at least 5 to 7 or for one or more than one parameter a score of 3. If this would have been confirmed, birds would have been immediately taken to the necropsy hall and sacrificed. A therapeutic approach was not followed due to the fact that therapeutic intervention would modify the outcome and possible interpretation of the experiment.

In the case of minor injuries affected birds would have been separated within the isolation room from the group and treated with a silver spray to protect the injured area and support the healing process. The birds were placed back in the group, if a clear recovery was visible.

If more birds would have been injured due to pecking, the light intensity would have been reduced to stop pecking.

If the group would have shown at least one of the following symptoms: depression, ruffled feathers, closed eyes, reluctant to move, huddling, birds would have been clinically observed at least two to three times/day, and the room temperature be raised.

### Virus and inoculum preparation

The vvIBDV strain 89163/7.3, used in this study, was kindly provided by N. Eterradossi, AFSSA, Ploufragan, France [18]. The preparation and the challenge dosage of vvIBDV with 10^3^ egg infectious dose (EID)_50_ /bird via eye drop were described previously [19]. The virus had been stored at -80°C.

### Histological investigations

Samples of the BF, CT and the middle region of the caecum were collected, fixed in 4% (w/v) phosphate-buffered formalin for 48 hours, embedded in paraffin, sectioned (2 μm) and further processed for histological examination following standard procedures as previously described [20]. Bursal follicular lesion scores were determined according to previously described scores (lesion scores: score 1 = 1–25%, score 2 = 26–50%, score 3 = 51–75% and score 4 = 76–100% of bursal follicles showing more than 50% lymphoid cell depletion [21]. CT lesions included edema, infiltration of plasma cells and heterophilic granulocytes.

Caecum lesions were characterized by the loss of epithelial integrity, edema, infiltration of plasma cells as well as heterophils [9, 22]. Total mucosal thickness, including the mucosal epithelium and lamina propria of the cecum were determined by morphometric analysis. The caecum mucosa was measured at 5 representative points in each caecum using ImageJ software (National Institutes of Health, USA), with the line tool calibrated from pixels to micrometers using the reference bar standard (calibration: 0.457 pixels/micrometers). The mean of mucosal thickness was calculated for six birds per group.

### Immunohistochemical staining of vvIBDV antigen

Sections of the BF, CT and the middle region of the caecum were prepared as previously described [20]. vvIBDV antigen was detected using a polyclonal rabbit anti-IBDV serum at a dilution of 1:5000 [21]. The secondary anti-rabbit IgG biotinylated antibodies and ABC reagent (Universal Vectastain ^®^Elite^®^ABC Kit, Vector Laboratories Inc., Wertheim-Bettingen, Germany) were used according to the manufacturer’s instructions [23]. DAB (DAB peroxidase substrate Kit, Vector Laboratories Inc.) was used to visualize the enzyme-linked complex. Sections were investigated by light microscopy. The antigen score of each group is based on the number of vvIBDV-antigen positive cells per field at a magnification of 200 x: 1 = 1–10, score 2 = 11–50, score 3 = 51–100 and score 4 = over 100 vvIBDV antigen-positive cells in 10 randomly selected microscopic fields per bird (n = 6/group).

### Mast and goblet cell staining

The preparation of the BF, CT and the middle of the caecum for mast and goblet cell staining was done as previously described [9]. Briefly, for the mast cell staining, sections of 2 μm were stained with 0.8% toluidine blue (Sigma Co., UK) for 2 min after the rehydration. Slides were washed with distilled water for 1 min, immediately dehydrated using 95% alcohol, and 100% alcohol for 2 min, cleaned with xylene and then mounted with neutral gums. For the goblet cells staining, sections were stained with 1% Alcian blue 8GS in PBS (Sigma Co., UK) for 15 min, rinsed for 5 min with distilled water, dehydrated with 95% alcohol and 100% alcohol for 2 min, respectively, cleaned with xylene and then mounted with neutral gums. The number of mast cells in the caecum was counted in five randomly selected fields per bird (n = 6/group) under the microscope (200 x magnification) and the goblet cells were counted per villus, from tip to the crypt and calculated as the mean number per villus.

### Detection of immune cells by immunohistochemistry

Samples of BF, CT and the middle region of the caecum were snap frozen in liquid nitrogen. Frozen sections of tissues of 4 μm were prepared. Immunohistochemical staining of immune cells was conducted according to the manufacturer’s instructions [23]. The following primary antibodies were used at the following work concentration of 0.05 μg/ml: anti-CD4, anti-CD8β, anti-Bu1, anti-KuL01 and anti-IgA (Southern Biotech, provided by Biozol, Eching, Germany). Secondary anti-mouse IgG biotinylated antibodies, ABC reagent (Vectastain ®Elite®ABC Kit, Vector Laboratories Inc., Wertheim-Bettingen, Germany), as well as DAB (DAB peroxidase substrate Kit, Vector Laboratories Inc.) were used according to the manufacturer's instructions [24]. Sections were examined via light microscopy. The lymphocyte populations and macrophages in the BF as well as the IgA-positive cell populations in the caecum were evaluated by counting the number of stained cells at a magnification of 200x in five randomly selected microscopic fields per bird (n = 6/group). The caecal Bu1+ and T LPL as well as KuL01+ cells were evaluated by counting the number of positive cells per three crypt regions at a magnification of 200x of five randomly selected fields per bird (n = 6/group) [24].

For the epithelial lymphocytes (IEL), the numbers of CD4+ and CD8ß+ cells were evaluated by counting the positive cells in the epithelial layer at a magnification of 200 × of five randomly selected fields.

### Gut microbiota composition

Microbiota composition was determined by sequencing of the V3/V4 variable region of 16S rRNA genes exactly as described previously [25]. The resulting sequences were classified by RDP Seqmatch with an OTU (operational taxonomic units) discrimination level set to 97% using Qiime software.

### vvIBDV-antibody detection by ELISA

Circulating anti-IBDV-specific IgG antibodies were detected by the commercially available enzyme-linked immunosorbent assay (ELISA) ProFLOK*®* IBD PLUS ELISA antibody test kit (Synbiotics Co., Kansas City, Mo.). Anti-IBDV-antibody titers were calculated based on the OD values and are presented as mean titer ± standard deviation (SD) per group.

### Experimental design

Two experiments were conducted in the present study.

All people who participated in the animal experiments were either veterinarians with over 10 years of experience in conducting animal experiments with birds or specifically trained by attending a FELASA C course, or were animal care takers, which were specialized in managing poultry under experimental conditions. All participants were specifically approved by the Lower Saxony State Office for Customer Protection and Food Safety (LAVES) to contribute to these animal studies.

#### Experiment 1

Forty-eight one-day-old commercial broiler chickens were raised at the Clinic for Poultry and were randomly divided into two groups (vvIBDV-inoculated group and virus-free control). At seven and 14 days post hatch (dph), sera were collected for maternally derived antibody (MDA) detection. Twenty-four chickens were inoculated with vvIBDV at the age of 15 dph with a dosage of 10^3^ egg-infectious dose (EID)_50_/bird via eye drop. Twenty-four chickens were kept as virus-free controls which received PBS. Clinical signs were monitored throughout the whole experiment. Six birds of each group were randomly selected and sacrificed at three, seven, 14 and 21 days post inoculation (dpi), when the experiment was terminated. Serum samples were collected for the detection of vvIBDV specific IgG antibodies by ELISA. BF was weighted to calculate the organ to body weight ratio. Pathological lesions were determined. Samples of BF, CT, as well as the middle of the caecum were formalin-fixed and sectioned for the detection of histopathological lesions. Samples of BF, CT and the middle region of the caecum were collected for immunohistochemical detection of vvIBDV-antigen, immune cells, mast and goblet cells. In addition, caecum content was collected at necropsy for gut microbiota composition analysis.

#### Experiment 2

Experiment 2 was partially a repeat of Experiment 1 with a total of thirty-six broiler chickens. Eighteen chickens were inoculated with vvIBDV at a dose of 10^3^ EID_50_/bird at 14 dph. The remaining eighteen chickens were kept as virus-free controls. Six broilers of each group were randomly selected and different to Experiment 1 necropsied at 10, 14 and 21 dpi, when the experiment was terminated. As in Experiment 1, serum samples were collected for MDA detection and at necropsy for the detection of vvIBDV specific IgG antibodies by ELISA. Parameters, which were investigated at necropsy in Experiment 2 as a repeat of Experiment 1 include: bursa/body weight ratio, histological lesions, viral-antigen detection, immune cell populations including T and B lymphocytes, mast cell, and goblet cells in the BF, CT, and caecum. Different to Experiment 1, gut microbiota composition of caecum content was only determined at 14 dpi.

In all experiments birds were randomly selected for necropsy (n = 6/group and time point in each experiment). They were stunned using blunt trauma, which was placed on the fronto-parietal region, and subsequently immediately killed by exsanguination, which was approved by the animal welfare committee of the Lower Saxony State Office for Customer Protection and Food Safety.

### Statistical analysis

Statistical analysis was performed using Statistix version 9.0 (Analytical software, Thallahassee, USA). Two Sample T test (two-tailed) was used to analyze the difference in the total mucosal thickness, and LPL immune cells between groups because data were normally distributed as tested by the Shapiro Wilk-Test. The Wilcoxon Rank Sum T test was used to analyze the differences in IEL, mast cells, and antibodies titer between groups at the indicated time points because data for these parameters were not-normally distributed as tested by the Sharpiro Wilk-Test. *P* < 0.05 was considered as statistically significant. Graphs were prepared with GraphPad v6 (Prism, LaJolla, USA).

## Results

### Clinical signs and tissue lesion development

vvIBDV was inoculated when MDA were below the breakthrough titer of the virus in both experiments. In Experiment 1 and 2, vvIBDV was inoculated at 15 and 14 days post hatch based on the break-through levels as calculated by the Deventer formula and published previously (data not shown) [26].

No chicken died due to vvIBDV-infection in either experiment, and none showed clinical signs including ruffled feathers, huddling, respiratory distress or diarrhea after virus-inoculation (S1 Table), which confirms previous experimental vvIBDV-infection studies in commercial broilers [19, 27].

Consistent with previous studies [27, 28], vvIBDV inoculation induced a significant increase in the bursa to body weight ratios (B/BW) at three dpi in comparison to the virus-free controls (S2 Table, *P* < 0.05). Afterwards starting at seven dpi, bursal atrophy was observed in vvIBDV-infected birds compared to virus-free controls. Comparable results were observed in Experiment 2, with a significant decrease in B/BW at 21 dpi in comparison to the virus-free control (S2 Table, *P* < 0.05). vvIBDV infection induced an increase in anti-IBDV IgG-specific antibodies in both experiments. A significant upregulation was observed in both experiments with ELISA-titers of log_10_ 3.5 ± 0.2 at seven dpi in Experiment 1 and 3.2 ± 0.2 in Experiment 2 at 10 days post hatch compared to virus-free controls in Experiment 1 (2.4 ± 0.8) and 2 (1.6 ± 0.8), respectively (*P* < 0.05).

A depletion of lymphoid cells in bursal follicles was observed microscopically throughout both experiments, and a trend of bursal recovery with beginning repopulation of follicles was observed starting at 21 dpi (S1 Fig). Bursa lesion scores were 4.0 ± 0.0 starting at three dpi in all vvIBDV-infected groups in both experiments (S3 Table).

**Fig 1:**
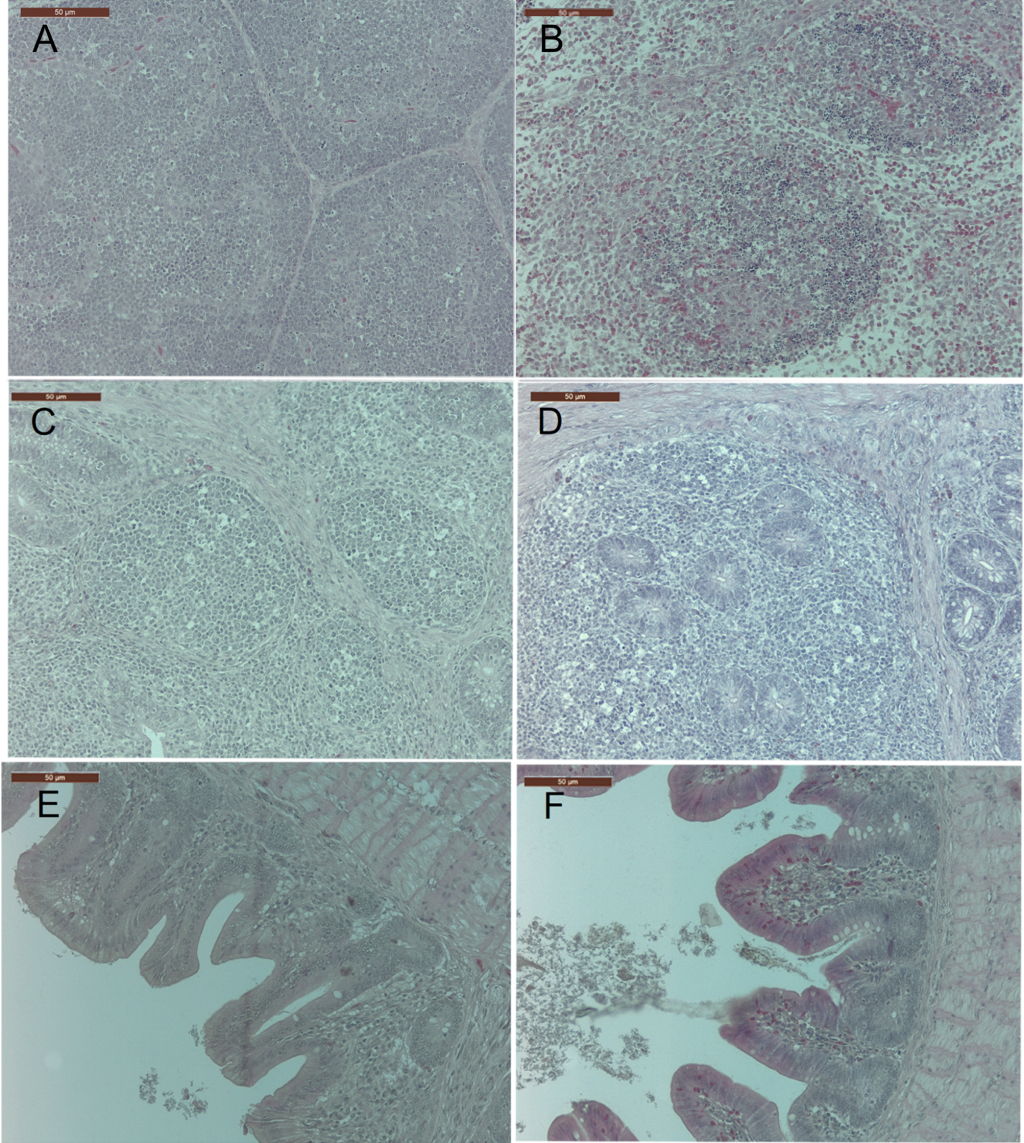
**vvIBDV infection led to histological lesions in the BF (A, B), CT (C, D) and caecum (E, F) (Experiment 1 as a representative experiment).** Control = virus-free control, vvIBDV = vvIBDV-infected group. A, C, E are representative sections from virus-free controls at three dpi, and B, D, F are representative sections from vvIBDV-infected birds at three dpi.

An infiltration of heterophils was observed in the BF of vvIBDV-infected birds at three dpi in Experiment 1 (Fig 1A and 1B). In the CT, vvIBDV-infected birds exhibited cellular destruction of germinal centers (Fig 1D), and a slight infiltration of heterophils in the submucosal area at three and seven dpi (Experiment 1), these lesions were not observed at later time points in either experiment. An infiltration of heterophils was also detected in the caecum of vvIBDV–inoculated birds at three and seven dpi (Experiment 1) in comparison to virus-free controls (Fig 1E and 1F). A significant decrease in the total mucosal thickness was observed at seven, 14 and 21dpi in the caecum of vvIBDV–inoculated birds compared to virus-free controls (Table 1, *P* < 0.05).

**Table 1:** Morphometric measurements of the caecum mucosa of virus-free and vvIBDV-inoculated birds. Results are expressed as mean± SD (μm) (Experiment 1).

Dpi	Average total mucosal thickness (μm)	
	Control	vvIBDV
3	245.6±67.2	230.6±25.6
7	342.3±48.9	213.2±35.8*
14	262.9±22.7	205.1±47.6*
21	283.6±32.3	216.3±25.2*

Dpi = days post inoculation; Control = virus-free control; vvIBDV = vvIBDV-infected group.

*indicates significant differences between groups at the indicated time points (*P* < 0.05, n = 6/group).

### Effect of vvIBDV on goblet and mast cells

**Fig 2:**
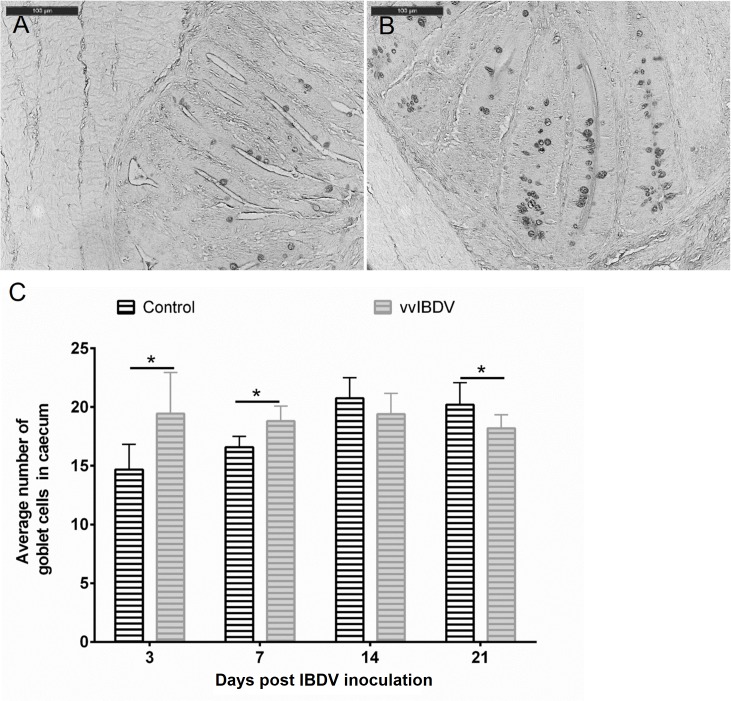
**Goblet cell staining in the caecum (A, B) of birds after three days post vvIBDV inoculation (Experiment 1) and average number of goblet cells at varies time points post virus-inoculation (C).** A, is the representative picture from virus-free controls, and B is the representative picture from a vvIBDV-infected bird. C is the summary of the number of goblet cells in the caecum in Experiment 1. * significantly different between groups at the indicated time points (Wilcoxon Rank Sum Test, *P* < 0.05). control = virus-free control, vvIBDV = vvIBDV-infected group.

Consistent with previous studies [9], an increase in the number of goblet cells in the out layer of the bursa was observed in the vvIBDV-infected chickens in comparison to virus-free controls in both experiments (data not shown). In the caecum, vvIBDV-infected birds showed a significant increase in the number of goblet cells at three, seven (Experiment 1, Fig 2) and 10 dpi (data not shown, Experiment 2) compared to virus-free controls (*P* < 0.05). While a significant lower number of goblet cells was observed at 21 dpi in the caecum of vvIBDV-infected birds compared to virus-free control (Fig 2 and S2 Fig, *P* < 0.05)

**Fig 3:**
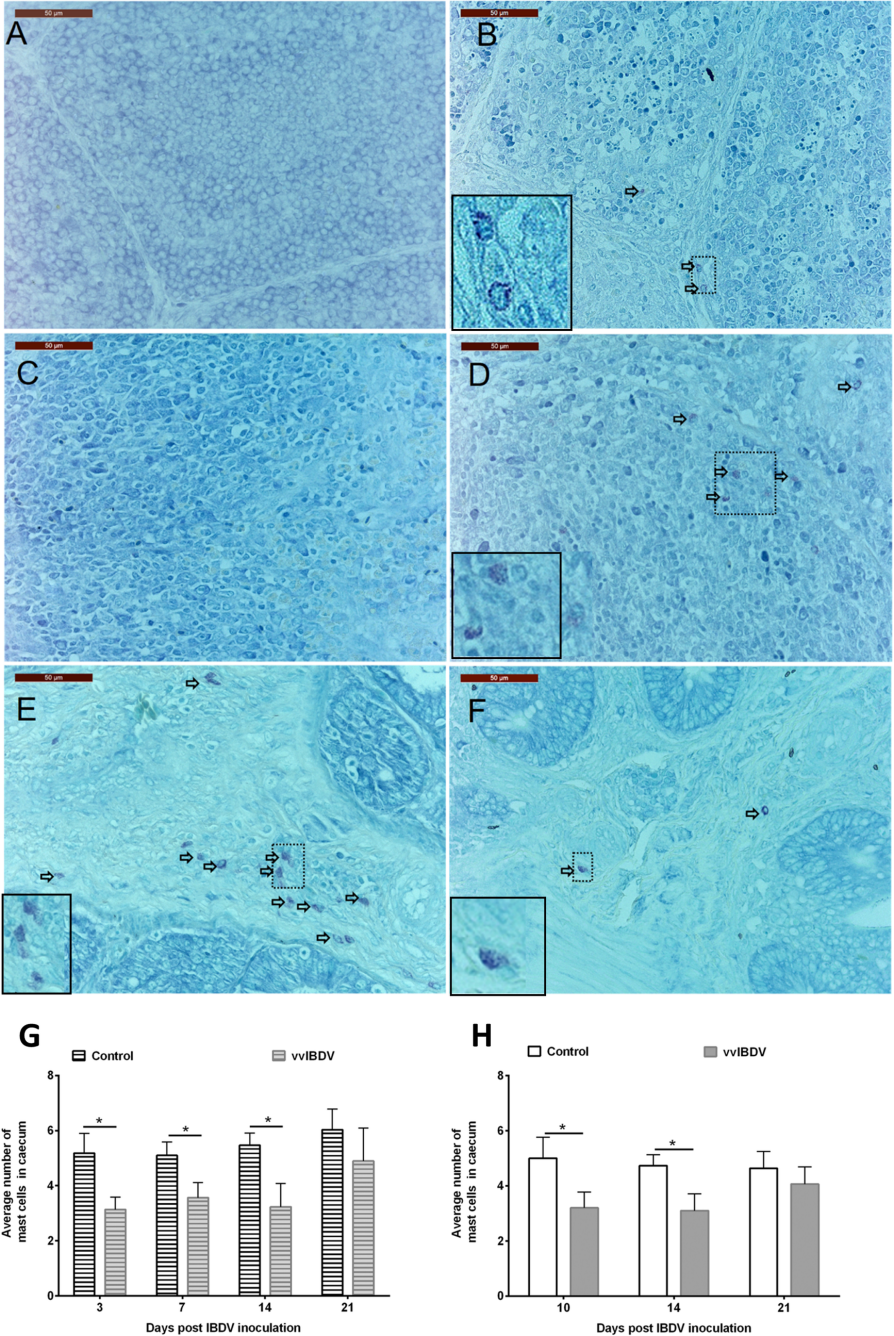
**Mast cell detection in the BF (A, B), CT (C, D) and caecum (E, F) of control (A, C, E) and vvIBDV-inoculated (B, D, F) birds after three days post vvIBDV inoculation (Experiment 1) and a summary of mast cell detection over time in the caecum of animals in Experiment 1 (G) and Experiment 2 (H).** Arrows indicate stained mast cells. Enlargements of indicated regions with mast cells are presented in figures B, D, E, F. *indicates significant differences between groups at the indicated time points (Two-sample T test, *P* < 0.05). Control = virus-free control, vvIBDV = vvIBDV-infected group. n = 6 per group.

Mast cells were frequently detected in the mucosal LP of the caecum, while they were rarely detected in the BF and CT of virus-free birds. The effect of vvIBDV on the mast cell numbers varied between different tissues. A transient increase of mast cell numbers was observed at three dpi in the BF and CT of vvIBDV-infected birds in comparison to virus-free controls in Experiment 1 (Fig 3A–3D). Compared to virus-free controls, a significant decrease in the number of mast cells in the caecum was observed at three, seven and 14dpi in Experiment 1 and at 10 and 14 dpi in Experiment 2 in the vvIBDV-infected birds (Fig 3E–3H, *P* < 0.05).

### vvIBDV antigen detection

**Fig 4:**
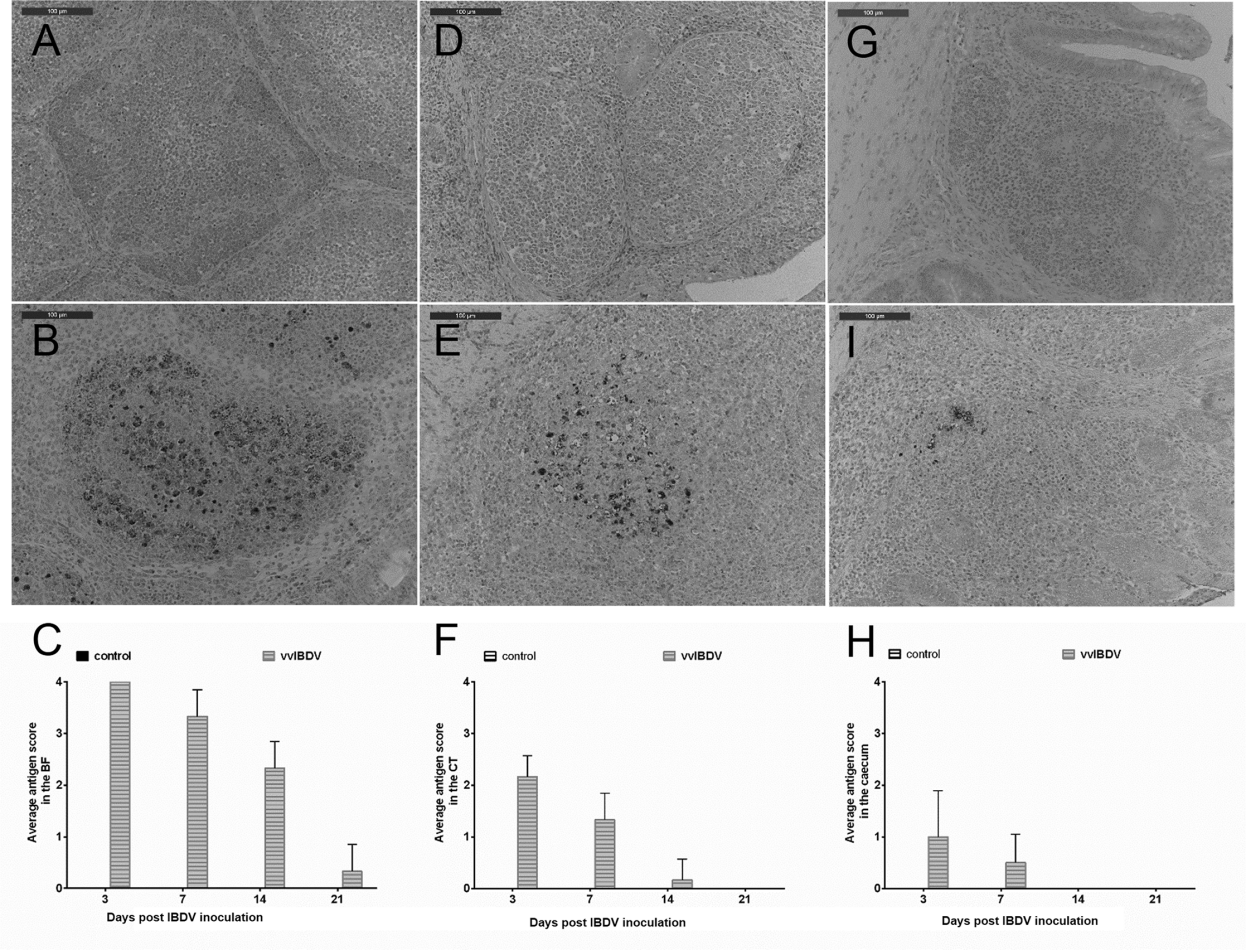
**Staining of IBDV-antigen in the BF (A, B, C), CT (D, E, F), and caecum (G, I, H) of chickens at three days post vvIBDV-inoculation (Experiment 1).** Control = virus-free control, vvIBDV = vvIBDV-infected group. A, D, G are sections from virus-free controls, and B, E, I are from vvIBDV-infected birds. C, F, H are the summary of antigen positive cells in the BF, CT and caecum, respectively.

All virus-free birds were negative for vvIBDV. Consistent with previous studies, vvIBDV replication was most vigorous in the BF [27, 28]. vvIBDV antigen was detected at high levels in the BF at three dpi (Experiment 1, Fig 4), and then the number of antigen-positive cells decreased over time. At 21 dpi nearly no IBDV-positive cells were detected in the BF in either experiment. vvIBDV-antigen positive cells were also observed at three and seven dpi in the CT and caecum and at 14 dpi in the CT of vvIBDV-infected birds in comparison to virus-free controls in Experiment 1 (Fig 4). No IBDV-positive cells were detected in the CT and caecum at 10, 14 and 21 dpi in Experiment 2. The score of vvIBDV antigen-positive cells was lower in these tissues compared to the BF.

### Effects of vvIBDV-infection on immune cells in the LP of the GALT

**Fig 5:**
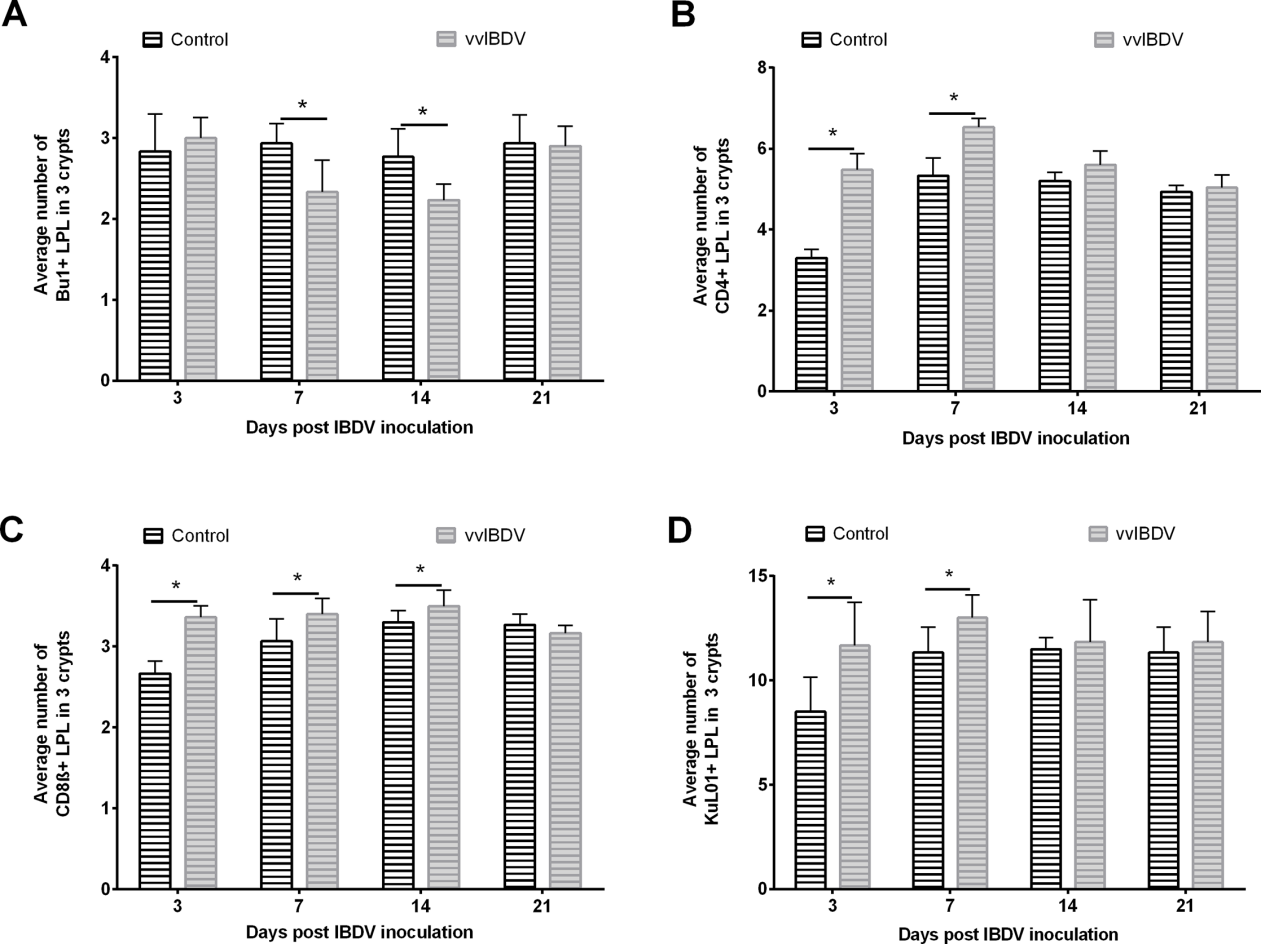
**Immunohistochemical detection of Bu1+ (A), CD4+ (B), CD8β+ (C), and KuL01+ (D) cells in the caecum of chickens at different days after vvIBDV-infection (Experiment 1).** *indicates significant difference between groups at the indicated time points (Two-sample T test (A) or Wilcoxon Rank Sum Test (B, C, D), *P* < 0.05). Control = virus-free control, vvIBDV = vvIBDV-infected group.

**Fig 6:**
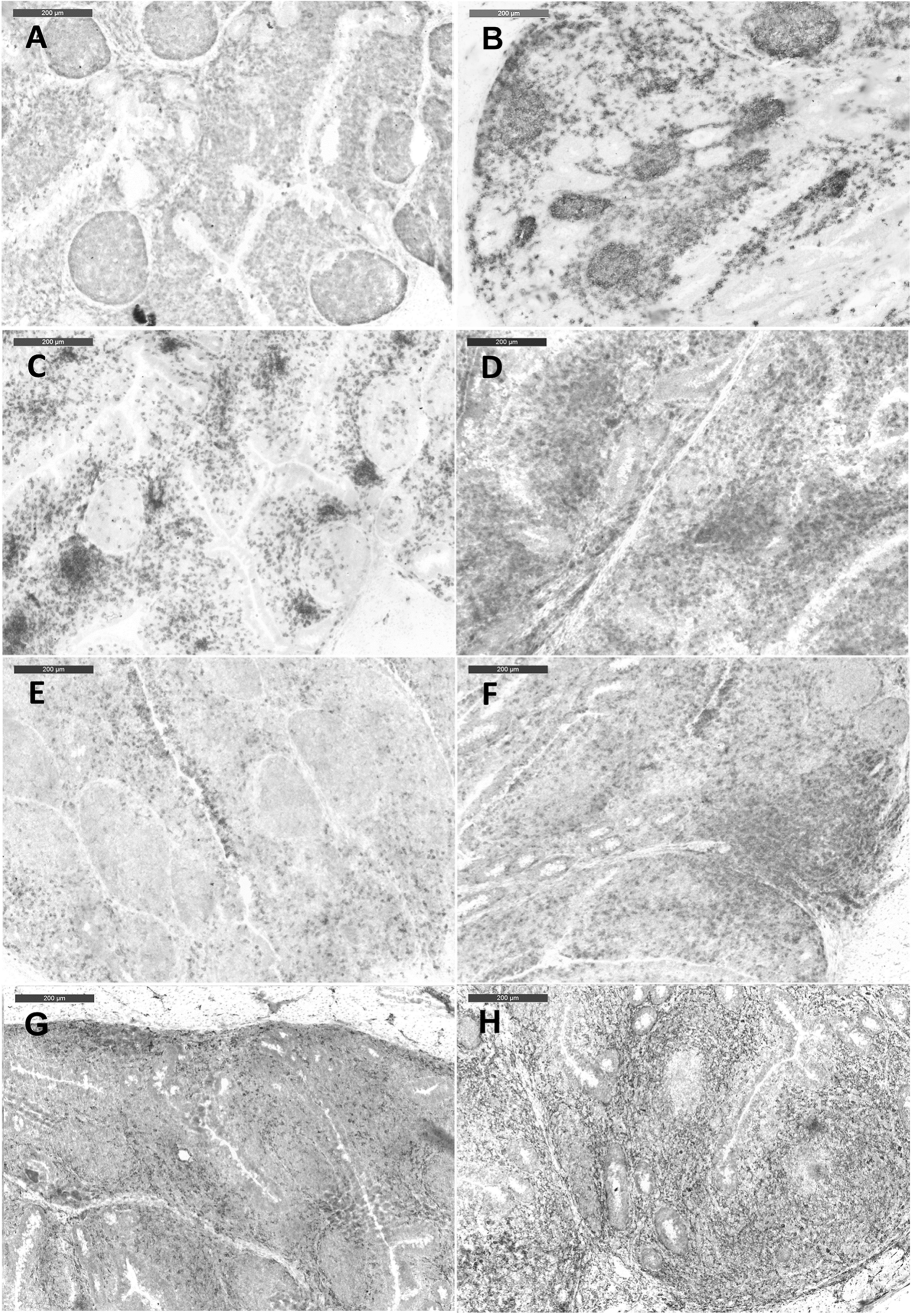
**Immunohistochemical detection of Bu1+ (A, B) CD4+ (C, D), CD8β+ (E, F), and KuL01+ (G, H) in the CT of chickens after three days post vvIBDV inoculation (Experiment 1).** A, C, E, G are from virus-free controls, and B, D, F, H are from vvIBDV-infected birds.

Consistent with previous studies, vvIBDV infection led to a depletion of B lymphocytes, a significant increase in T lymphocytes, as well as transient infiltration of macrophages at three and seven dpi in the BF (*P* < 0.05) [21]. In the caecum, vvIBDV-infected birds showed a significant decrease in the number of LP B lymphocytes at seven and 14 dpi compared to virus-free controls in Experiment 1 (Fig 5A and S3 Fig, *P* < 0.05), and this significant difference was also observed at 10 and 14 dpi in Experiment 2 (*P* < 0.05). Compared to virus-free controls, vvIBDV-infected birds had a reduced number of B cells and smaller sizes of germinal centers in the CT throughout both experiments (Fig 6B). The decrease in the number of LP B lymphocytes in the caecum coincided with a significant decrease in the number of IgA secreting cells in the caecum in the area of the lamina propria in Experiment 1 of vvIBDV-infected birds in comparison to virus-free controls (Table 2, S4 Fig). No IgA-positive cells were detectable in the epithelium.

**Table 2:** Average number of IgA+ cells in the in the lamina propria of the caecum (Experiment 1).

Dpi	Average number of IgA+ cells in the caecum ± SD	
	Control	vvIBDV
**3**	19.6±3.8	12.2±3.0*
**7**	31.6±3.8	15.9±2.9*
**14**	39.6±10.7	28.7±7.4
**21**	52.8±10.1	35.1±12.3*

Dpi = days post inoculation; Control = virus-free control; vvIBDV = vvIBDV-infected group.

*indicates significant differences between groups at the indicated time point (*P* < 0.05, n = 6/group).

An increase in the number of CD4+ and CD8ß+ LPL in the caecum was detected in both experiments. This increase was significant at three, seven and 14 dpi when comparing vvIBDV-infected birds and virus-free controls in Experiment 1 (Fig 5B and 5C, *P* < 0.05). Comparable results with a significant increase in the number of CD4+ LPL at 10 dpi were observed in the caecum of vvIBDV-infected birds compared to virus-free controls in Experiment 2 (*P* < 0.05). Similar results were observed in the CT with an infiltration of T cells in the submucosal area at three dpi (Fig 6D and 6F).

A significant increase in the number of macrophages was observed at three and seven dpi in the caecal LP of vvIBDV-infected birds compared to virus-free controls (Fig 5D, *P* < 0.05). An increase in the amount of macrophages was also induced by vvIBDV in the CT at three and seven dpi, but not at later time points (Fig 6H).

### Effects of vvIBDV-infection on IEL in caecum

**Fig 7:**
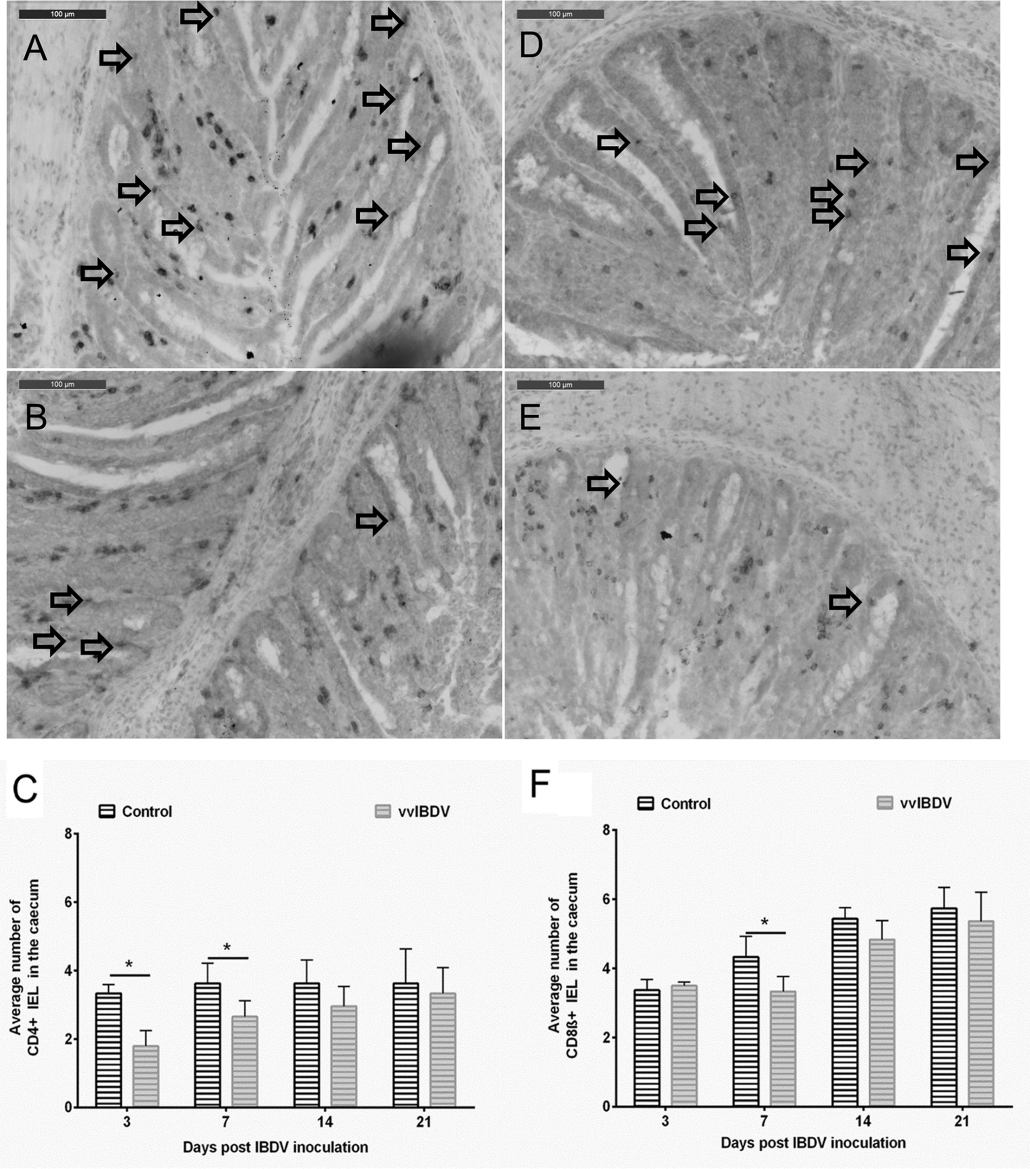
**Average number of CD4 (A, B, C) and CD8β-positive (D, E, F) cells located within the epithelia (IEL) of the caecum of chickens at seven (A, B, D, E) or different days post vvIBDV inoculation (C, F) (Experiment 1).** *indicates significant difference between groups at the indicated time points (Wilcoxon Rank Sum T test, *P* < 0.05). Control = virus-free control, vvIBDV = vvIBDV-infected group. Arrows indicate IEL cells, n = 6 per group.

IEL B cells were rarely detectable in non-as well as infected birds. A significant decrease in the number of CD4+ IEL was observed at three and seven dpi in the caecum of vvIBDV-infected birds compared to virus-free controls in Experiment 1 (Fig 7A–7C; *P* < 0.05). vvIBDV-infected birds also had a significant decrease in CD8ß+ IEL numbers at seven dpi in the caecum compared to virus-free controls (Fig 7D–7F; *P* < 0.05).

### Effect of vvIBDV on caecum microbiota composition

**Fig 8:**
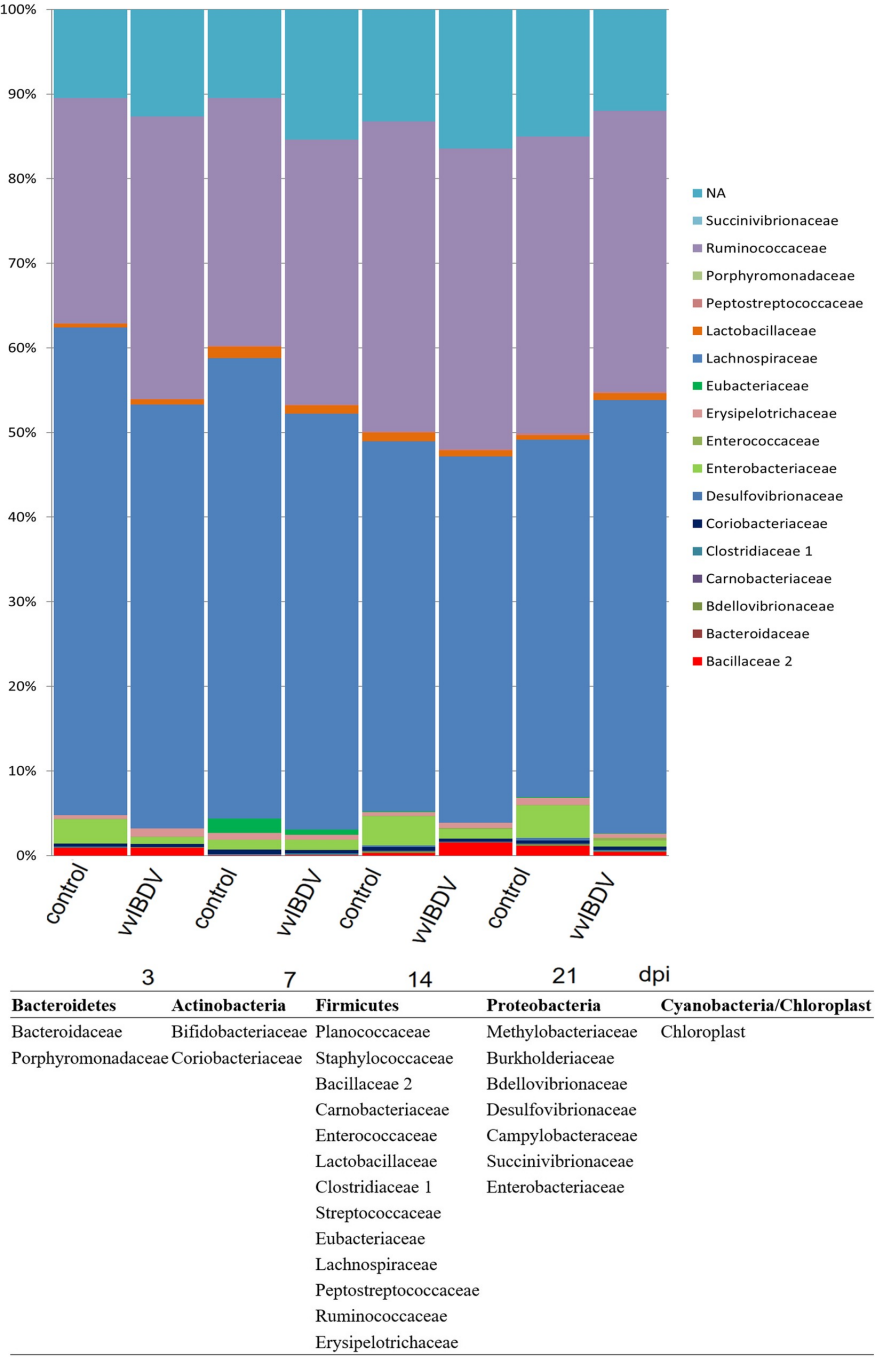
Bacterial communities of caecal samples from chickens at the family level (Experiment 1). Data were analyzed using QIIME. The *x*-axis represents the groups at different days post inoculation (dpi) and the *y*-axis represents the relative abundance of sequences. Control = virus-free control, vvIBDV = vvIBDV-inoculated group. NA = not analysed.

**Fig 9:**
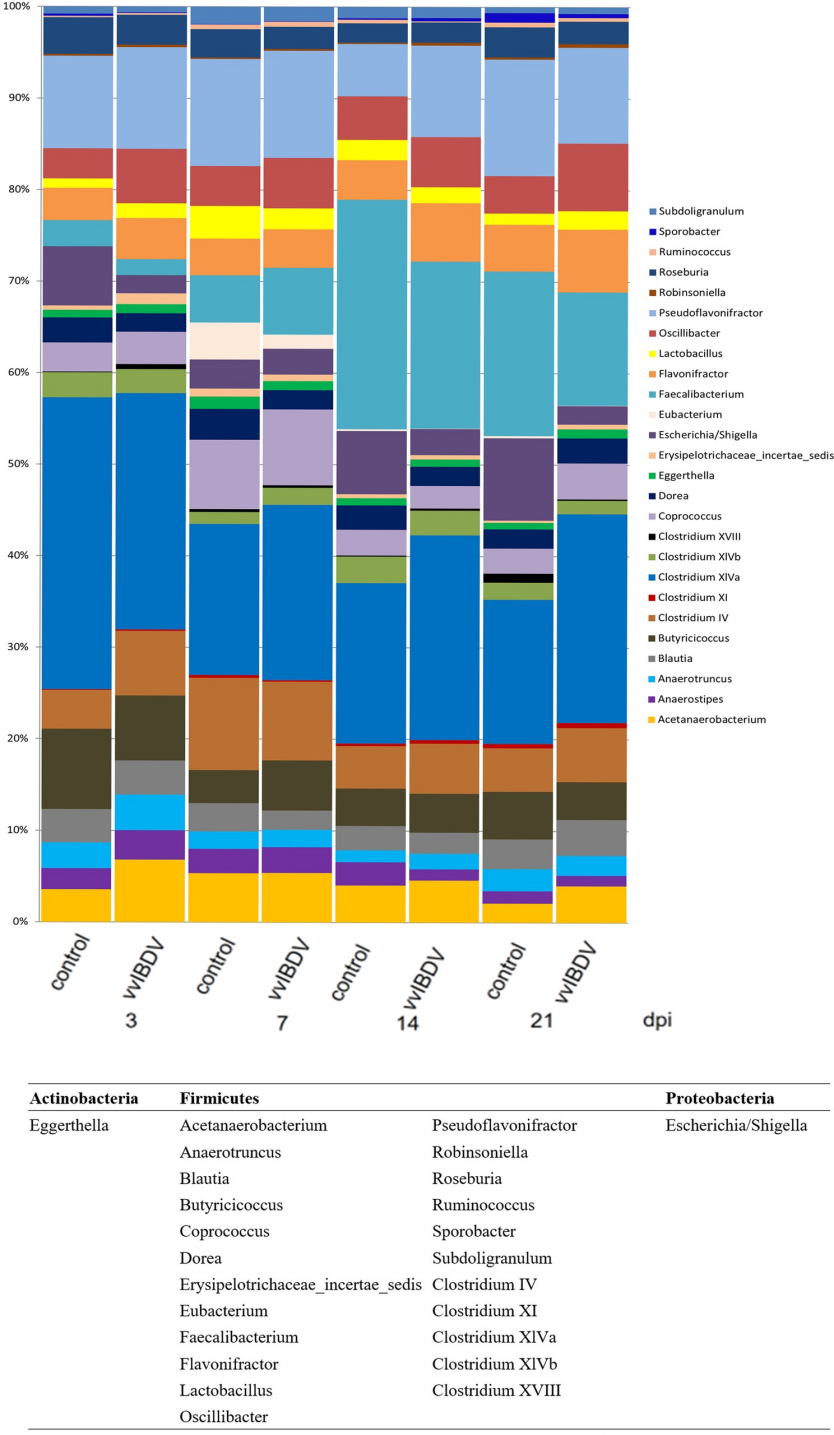
Bacterial communities of caecal samples from chickens at genus level (Experiment 1). Data were analyzed using QIIME. The *x*-axis represents the groups at different days post inoculation (dpi) and the *y*-axis represents the relative abundance of sequences. Control = virus-free control, vvIBDV = vvIBDV-inoculated group. NA = not analysed.

To obtain a deeper insight into the changes occurring to the caecal microbiota during vvIBDV infection, we investigated the caecal content at three, seven, 14 and 21 dpi in Experiment 1 and 14 dpi in Experiment 2. The sample with the lowest coverage was characterized by 3677 sequences, and 10283 sequences were available for the sample with the highest coverage. Representatives of nine phyla were detected at all the investigated time points. Independent of vvIBDV infection, the majority (over 95%) of microbiota was formed by representatives of *Firmicutes*, *Proteobacteria*, *Acitinobacteria* and *Bacteroidetes* (Figs 8 and 9). The relative representation of individual phyla in the caecal samples remained stable with *Firmicutes* forming more than 90% of the microbiota between 18 dph to 36 dph [29].

A more detailed analysis was performed on the family and genus level. At the family level, the majority of bacteria were *Lachnospiraceae* and *Ruminococcaceae* in both vvIBDV-infected and virus-free control groups. Independent of vvIBDV inoculation, the abundance of *Lachnospiraceae* decreased over time. It ranged from 54.4% at 18 dph to 42.2% at 36 dph. *Ruminococcaceae* showed a reverse trend with an abundance ranging from 25.6% at 18 dph to 42.2% at 36 dph (Fig 8). At the genus level, the abundance of *Faecalibacterium* increased ranging from 0.5% at 18 dph to 13.2% at 29 dph, and decreased afterwards to 9.3% at 36 dph (Fig 9).

vvIBDV inoculation modified the gut microbiota. Independent of age, vvIBDV inoculation led to a lower abundance of *Clostridium XlVa* at three dpi, which was followed by a higher abundance at seven and 21 dpi compared to virus-free controls (Fig 9). A higher abundance of *Faecalibacterium* at seven dpi, but a lower abundance at 14 and 21 dpi was observed in vvIBDV-infected birds in comparison to virus-free controls (Fig 9). There was also a decrease in abundance of *Escherichia/Shigella* detected at three, 14 dpi and 21 dpi in vvIBDV-infected birds compared to virus-free controls (Fig 9), confirming the decrease in the abundance of *Enterobacteriaceae* in the vvIBDV-infected birds compared to virus-free controls (Fig 8). Similar observations were made in Experiment 2 at 14 days pi.

## Discussion

Gut health is a very important aspect in poultry production and significantly contributes to the overall health and performance of a flock [30]. If the gut immunity as well as the mucosal intestinal barrier is disturbed, this may have a severe impact on the bird’s development and may enhance the risk for not only gut but also systemic infections [31]. Immunosuppressive diseases may influence the development of the gut immunity and possible the microbiota composition and subsequently modify the intestinal barrier [32, 33]. Neither the effect of vvIBDV infection on the GALT nor the possible correlation to the gut microbiota composition has been investigated so far. In the present study, commercial broiler chickens were infected with vvIBDV at 14 (Experiment 2) or 15 (Experiment 1) days post hatch, when MDA had reached the break-through levels of the virus. vvIBDV-infection was confirmed by viral antigen detection in the BF, and bursal atrophy with a depletion of B lymphocytes. Seroconversion was detected in vvIBDV-infected birds starting at seven dpi. Birds showed beginning recovery of microscopical lesions in the BF at 21 dpi.

In addition to the changes in the BF, detectable histopathological lesions were also observed in the CT and caecum of vvIBDV-infected birds. These findings coincide with the presence of virus antigen. CT and caecum showed structural recovery starting at 14 dpi. Viral clearance and recovery occurred faster in the CT and caecum compared to the BF. IBDV initially replicates in lymphocytes and macrophages in the gut intestine [34, 35]. It reaches the liver and enters the bloodstream leading to a primary viremia within 11 hours post infection. The virus starts replicating in proliferating B lymphocytes of the BF [35, 36]. Afterwards it migrates into the different tissues via blood circulation, causing secondary viremia [8]. In the present study, we observed the viral antigen positive cells in the germinal centers together with lesions from three to seven dpi. We speculate that the lesion in the CT and caecum are due to the secondary viraemia [37].

Gut associated mucosal immunity is important as a first barrier of host defense against pathogen invasion. An increase in the number of mast cells was observed in the BF and CT during the acute phase of the disease what confirms previous studies showing IBDV infection may affect the number and morphology of mast cells [9, 38]. Our method does not allow the differentiation if this was an increase in absolute numbers or just a change in relative cell numbers due to the IBDV-mediated B cell depletion. However Wang et al. [38] also demonstrated by using a comparable staining method a mast cell number increase in the thymus, a tissue, which is not as severely affected by B cell depletion as the bursa, providing circumstantial evidence that changes in mast cell numbers may not only be due to B cell depletion. vvIBDV infection also led to a significant decrease in number of mast cells in the caecum. We propose that this decrease contributes to a compromised gut mucosal immunity caused by vvIBDV infection [39]. A significant increase in the number of goblet cells was observed in the caecum of vvIBDV-infected birds compared to virus-free controls (*P* < 0.05). Goblet cells, together with epithelial cells and macrophages are regarded as the major cellular constituents of the innate defense system [40]. It was demonstrated that various enteric infections including bacteria and viruses are associated with an alteration of the goblet cell response [41–43]. Goblet cell hyperplasia was suggested to be controlled by immunological mechanism during infection [44, 45]. vvIBDV-infection may also interfere with the function of goblet cells [39].

A significant increase in the number of T cells was observed in the BF and CT, as well as in the LP of the caecum of vvIBDV-infected birds compared to virus-free controls (*P* < 0.05). It has been shown that T lymphocytes infiltrate into the LP of the gut after various enteric infections, such as with rotavirus [46] or *Salmonella* Enteritidis [47]. Previous studies had already demonstrated that an infiltration of T cells into the BF starts at the early stage of IBDV-infection [48]. Cytotoxic T lymphocytes were suggested to play a role in the clearance of IBDV, but also to contribute to lesion development in the bursa [49, 50]. Interestingly also the number of T LPL increased in the caecum of vvIBDV-infected birds, which suggests that the T cells are not only activated in the BF but also in the gut. However, vvIBDV infection led to a significant decrease in the number of CD4+ IEL at three and seven and CD8ß+ IEL at seven dpi (*P* < 0.05). The increase in T LPL might be due to the movement of CD4+ and CD8ß+ lymphocytes from the intraepithelial to the submucosal area. LPL and IEL are two distinct components of the GALT. Based on our investigation there is not clear correlation between the increase in T LPL and IBDV-antigen, as there was no detectable IBDV-antigen in the LP.

A decrease in the number of IgA+ secreting cells was observed in the caecum of vvIBDV-infected birds compared to virus-free controls. IgA is the most common immunoglobulin in the mucosal tissue, being an important line of the immunological defense against invading enteric pathogens. In addition, IgA regulates the ecological balance of the microbiota and has a fundamental role in mucosal homeostasis [51]. This detected decrease in IgA+ cells may be due to direct infection of these cells by IBDV. But older studies suggested that IBDV may target receptors mainly presented on the surface of IgM-bearing cells. vvIBDV-exposure did not reduce the levels of total serum IgA, IgG, and IgM, nor did it affect IgG and IgA B-cells in the spleen, while the caecum was not investigated [52]. Therefore, we speculate that the decrease in IgM+ B cells in the BF and CT possibly led to the subsequent reduction of IgA+ secreting cells in the caecum. Further studies should be conducted to determine if local and systemic IgA-levels may be reduced under comparable experimental conditions. Overall, this reduction in IgA+ cells together with the decrease in the number of mast cells as well as T IEL may result in insufficient protection against other pathogens, such as Salmonella [53], or *Escherichia coli* [54]. Early findings indicated that IBDV-induced humoral immunity suppression led to failure of seroconversion to other pathogens including infectious bronchitis virus (IBV) [55], chicken infectious anemia virus (CIAV) [56].

The gastrointestinal tract represents one of the primary sites of exposure to pathogens [57]. There is limited literature on dysbiosis caused by viruses. Recent studies targeted at the influence of the human immunodeficiency virus (HIV) as well as the simian immunodeficiency virus (SIV) on the gut microbiota and showed a selective enrichment of few phenotypes in the gut microbiota after viral infection [58, 59]. Interestingly, we observed a higher abundance of *Ruminococcaceae* and *Desulfovibrionacea*e in vvIBDV-infected birds compared to virus-free control. Similar finding were observed in HIV chronically infected patients [58, 59]. It was also shown that HIV infection leads to a lower level of local IgA. This possibly contributes to HIV infection-associated enhanced microbial translocation, which may lead, in turn, to a chronic state of immune activation as noted in many HIV patients [60]. HIV targets the CD4+ cells, while IBDV targets B cells. However, both virus infections lead to a decrease in the number of IgA+ cells in the intestine. We may speculate the decrease in IgA might attribute to the dysbiosis of gut microbiota.

In the present study, vvIBDV-infected birds had a lower abundance of *Clostridium XIVa* at three dpi compared to virus-free birds. This trend reversed between seven and 21dpi. An increase in the abundance of *Faecalibacterium* was observed at three and seven dpi in the vvIBDV-infected birds compared to virus-free controls, while this trend also reversed starting at 14 dpi. The acute phase of IBDV infection is between three to five dpi, which includes the peak of IBDV replication, and a strong inflammatory response with a ‘cytokine storm’. After the acute phase, all these reactions decrease over time, possibly coinciding with modification in the gut microbiota composition. Previous studies showed that *Clostridium spp*. are strong inducers of colonic T regulatory (Treg) cells [61]. Treg cells are primary mediators in maintaining the immune homeostasis and play a critical role in the suppression of extensive intestinal inflammation. In inflammatory bowel disease also a decrease in the abundance of *Clostridium XIVa* and *Faecalibacterium* had been observed [62–64]. Therefore, the decrease in the abundance of *Clostridium* XIVa at three days post vvIBDV infection might suggest that vvIBDV interferes with the delicate balance of gut mucosal immunity and may support harmful intestinal inflammation. The role of *Faecalibacterium* is unknown in chickens. In human studies, it was demonstrated that *Faecalibacterium prausnitzii* is a sensor and a marker of human health [63]. Intestinal disorders, such as inflammatory bowel disease [65] and colorectal cancer [66] are associated with a diminished abundance of *Faecalibacterium prausnitzii*. If this observation can be transferred to chicken, our data provides circumstantial evidence that changes in the abundance of *Faecalibacterium* for example through vvIBDV infections are an indicator for intestinal disorders.

In conclusion, this study shows for the first time the influence of vvIBDV on the gut associated immune system and the microbiota composition. These results may help to understand the far-reaching consequences of immunosuppressive diseases in poultry. The change of the microbial populations correlated well with changes in immune cell populations such as mast cells, B cells especially IgA+ cells in the LP of the caecum and CT of vvIBDV-infected birds. Due to the complexity of the viral pathogenesis and the GALT system of the host, it is difficult to pinpoint the exact mode of action of the virus on the microbiota. It is not clear if there were direct or indirect effects of the virus. This has to be evaluated further to be able to improve chicken’s health in the field in the future.

## Supporting information

S1 Fig **Histological bursa lesions of virus-free control (A, C, E, G) and vvIBDV-inoculated (B, D, F, H) chickens at three, seven, 14 and 21 dpi.** Arrows indicate beginning recovery in some bursa follicles.
(TIF)

S2 Fig **Goblet cell staining in the caecum of virus-free control (A) and vvIBDV-inoculated (B) birds after 21 days post virus-inoculation (Experiment 1)**.
(TIF)

S3 Fig **Immunohistochemical detection of Bu1+ (A, B), CD4+ (C, D), CD8β+ (E, F), and KuL01+ (G, H) cells in the caecum of virus-free control (A, C, E, G) and vvIBDV-inoculated chickens after three days post virus-inoculation (Experiment 1).** Arrows indicate the positive immune cells in the lamina propria of the caecum.
(TIF)

S4 Fig **Immunohistochemical detection of IgA in the caecum of virus-free control (A) and vvIBDV-inoculated (B) chicken after three days post virus-inoculation (Experiment 1).** Arrows indicate IgA positive cells in the lamina propria of the caecum.
(TIF)

S1 Table Clinical scoring of virus-free control and vvIBDV-inoculated birds during Experiments 1 and 2. (DOC)

S2 Table Bursa to body weight ratio of chicken after vvIBDV inoculation. Dpi = days post inoculation; Control = PBS-inoculated control; vvIBDV = vvIBDV-infected group. *letter indicates significant differences between groups at the indicated time point (*P* < 0.05, n = 6).
(DOCX)

S3 Table Bursa lesion score after vvIBDV-inoculation (Experiment 1 as a representative experiment). dpi = days post inoculation; control = PBS-inoculated control; vvIBDV = vvIBDV-infected group. *indicates significant differences between groups at the indicated time point (*P* < 0.05, n = 6/group).
(DOCX)
