# Supplementary figures and images for: Infectious bursal disease virus infection leads to changes in the gut associated-lymphoid tissue and the microbiota composition
Source: PLoS One. 2018 Feb 1;13(2):e0192066. doi: 10.1371/journal.pone.0192066 (PMC5794159; doi:10.1371/journal.pone.0192066)

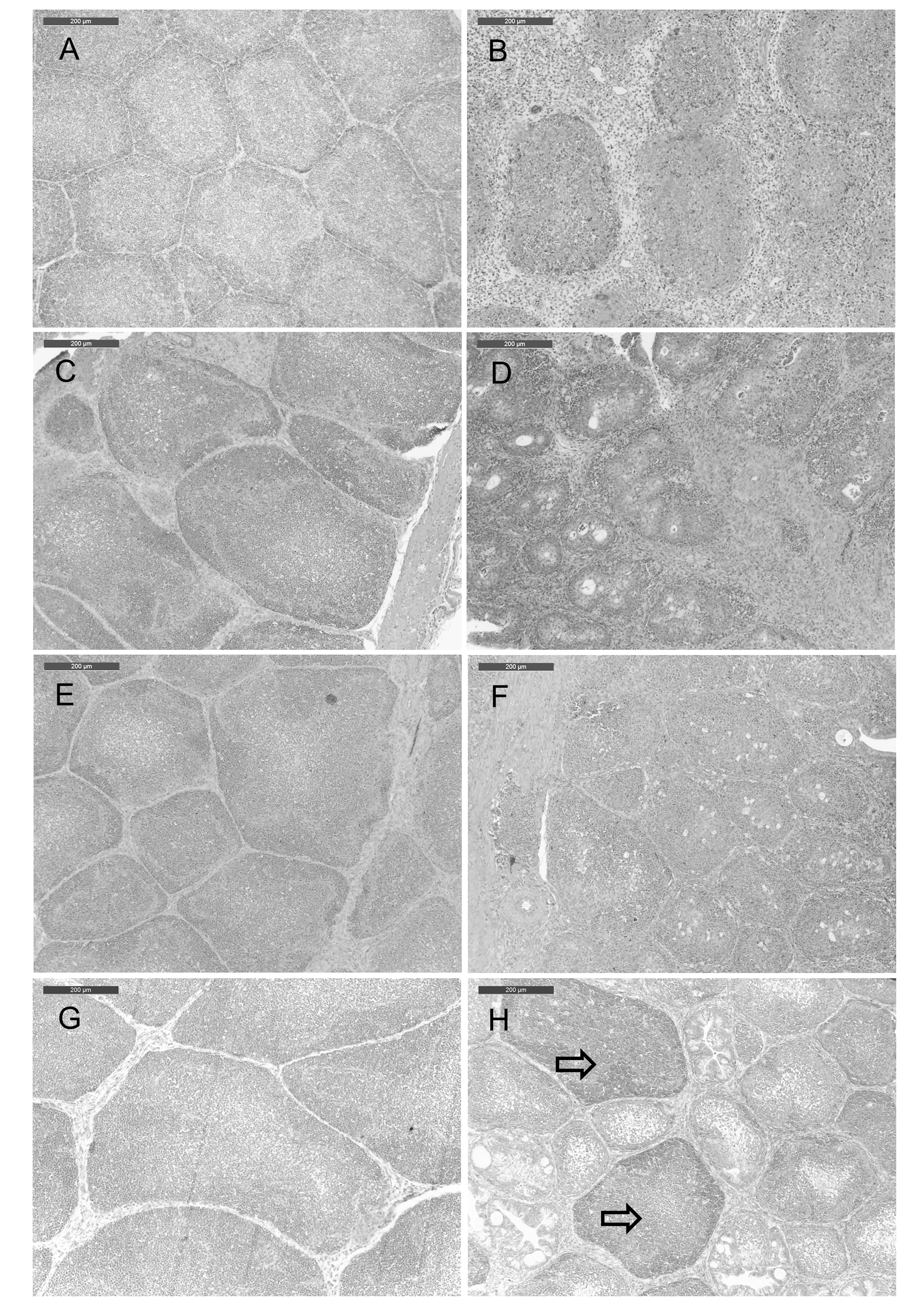

Supplement: S1 Fig — Histological bursa lesions of virus-free control (A, C, E, G) and vvIBDV-inoculated (B, D, F, H) chickens at three, seven, 14 and 21 dpi. Arrows indicate beginning recovery in some bursa follicles. (TIF) [file pone.0192066.s001.tif]

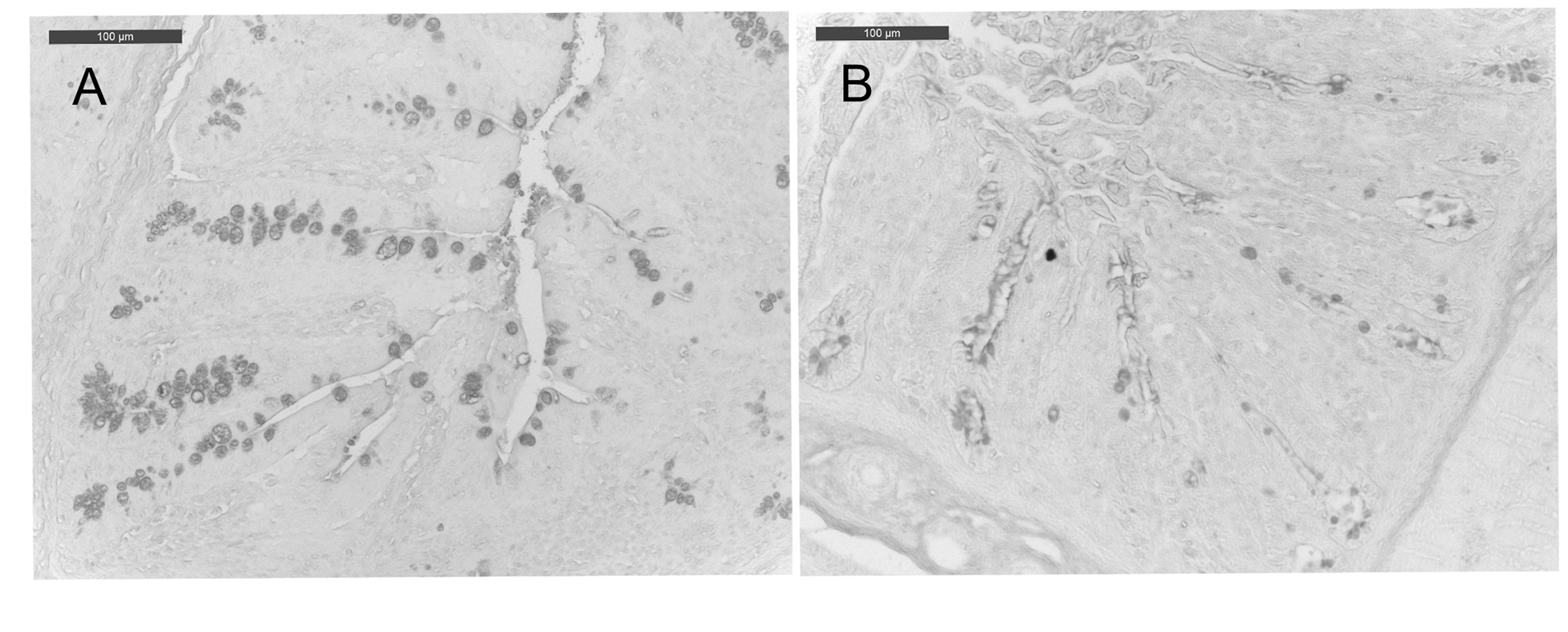

Supplement: S2 Fig — Goblet cell staining in the caecum of virus-free control (A) and vvIBDV-inoculated (B) birds after 21 days post virus-inoculation (Experiment 1). (TIF) [file pone.0192066.s002.tif]

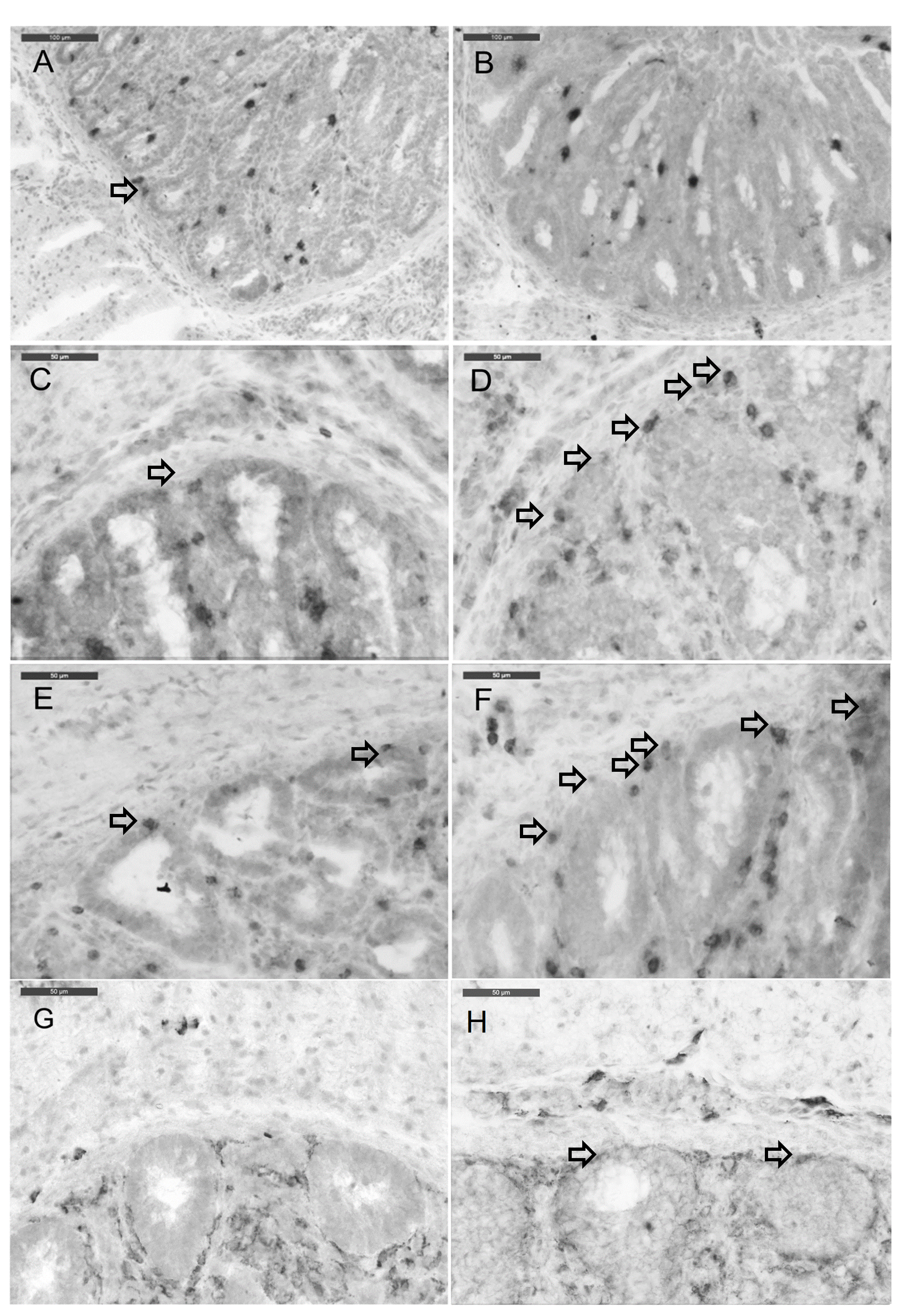

Supplement: S3 Fig — Immunohistochemical detection of Bu1+ (A, B), CD4+ (C, D), CD8β+ (E, F), and KuL01+ (G, H) cells in the caecum of virus-free control (A, C, E, G) and vvIBDV-inoculated chickens after three days post virus-inoculation (Experiment 1). Arrows indicate the positive immune cells in the lamina propria of the caecum. (TIF) [file pone.0192066.s003.tif]

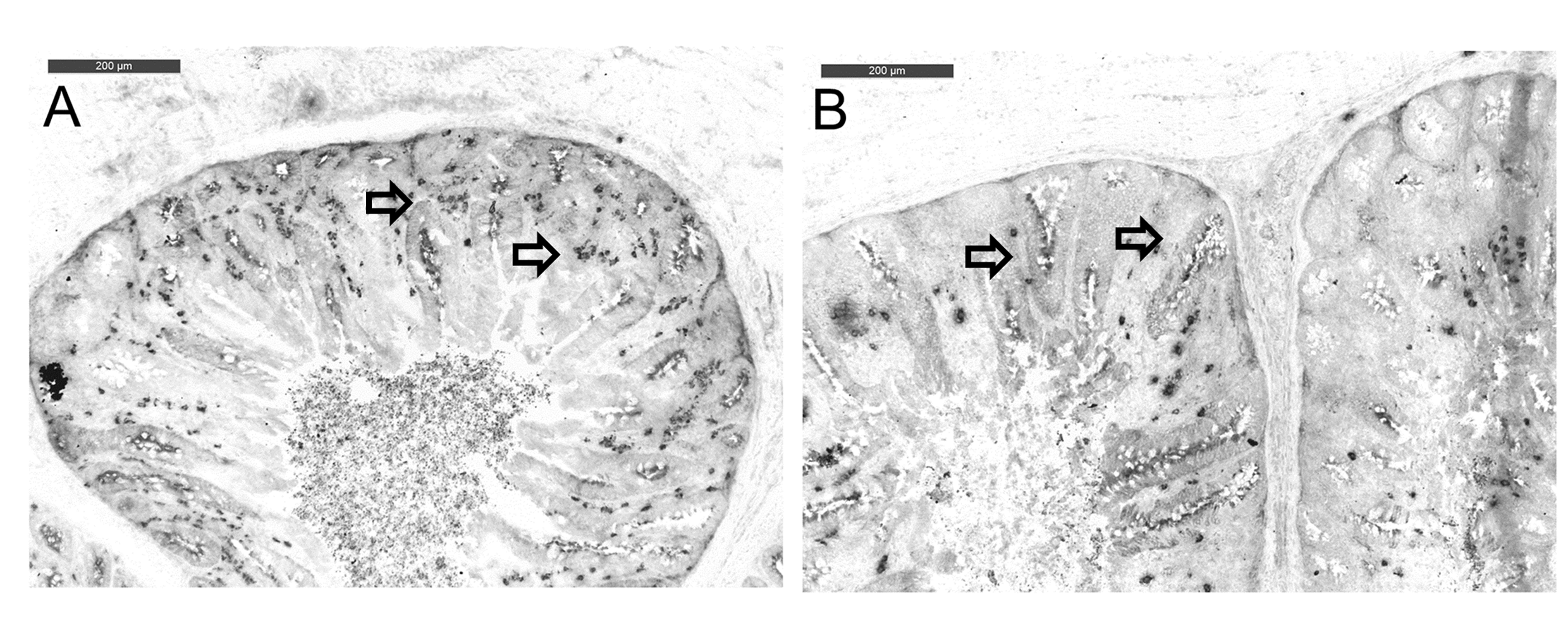

Supplement: S4 Fig — Immunohistochemical detection of IgA in the caecum of virus-free control (A) and vvIBDV-inoculated (B) chicken after three days post virus-inoculation (Experiment 1). Arrows indicate IgA positive cells in the lamina propria of the caecum. (TIF) [file pone.0192066.s004.tif]
