# Supplementary material for: Infectious bursal disease virus infection leads to changes in the gut associated-lymphoid tissue and the microbiota composition
Source: PLoS One. 2018 Feb 1;13(2):e0192066. doi: 10.1371/journal.pone.0192066 (PMC5794159; doi:10.1371/journal.pone.0192066)
Supplement: S1 Table — (DOC) [file pone.0192066.s005.doc]

**S1 Table. Clinical scoring of virus-free control and vvIBDV-inoculated birds during Experiment** 1 and 2.

| Experiments | Number of animals/group | Total number of animals with clinical anomalies | Average clinical scores/investigated parameter during the experimental time period | | | | | |
| --- | --- | --- | --- | --- | --- | --- | --- | --- |
|  |  |  | Abnormalities in breathing and /or excrements | Injuries | Changes in conjunctiva | Lesions at the blood removal site | Feed and water uptake | Movement |
| Exp. 1 | 24/control | 0 | 0 | 0 | 0 | 0 | 0 | 0 |
|  | 24/vvIBDV | 0 | 0 | 0 | 0 | 0 | 0 | 0 |
| Exp. 2 | 18/control | 0 | 0 | 0 | 0 | 0 | 0 | 0 |
|  | 18/vvIBDV | 0 | 0 | 0 | 0 | 0 | 0 | 0 |

Abnormalities in breathing and /or excrements: 0=normal, 1=slightly exacerbated and excrements normal, 2= moderately exacerbated and/or areas with excrements around the cloaca, 3= highly exacerbated and massive diarrhea; Injuries: 0= no injury , 1= injury (< 2cm of diameter), 2= bigger injury (> 2cm of diameter), 3= various injured skin areas or fractures; changes in conjunctiva: 0= no changes macroscopically detectable, 1= slightly red/swollen, 2= moderately red/swollen, ocular discharge on one side, 3= highly red/swollen, ocular discharge on both sides; lesions at the blood removal site:0= normal without lesions, 1= hematoma > size of a cherry pit, 2= hematoma > size of a table tennis ball, 3= non stoppable bleeding; feed and water uptake: 0= normal, 1= slightly reduced feed and water uptake, 2= moderately reduced feed and water uptake, 3= highly reduced feed and water uptake; Movement: 0= normal, 1= slightly reduced, 2= moderately reduced, 3= highly reduced or no moving of individual animals. control=virus-free control; vvIBDV=vvIBDV-inoculated group.
