# Supplementary material for: Infectious bursal disease virus infection leads to changes in the gut associated-lymphoid tissue and the microbiota composition
Source: PLoS One. 2018 Feb 1;13(2):e0192066. doi: 10.1371/journal.pone.0192066 (PMC5794159; doi:10.1371/journal.pone.0192066)
Supplement: S2 Table — Dpi = days post inoculation; Control = PBS-inoculated control; vvIBDV = vvIBDV-infected group. *letter indicates significant differences between groups at the indicated time point (P < 0.05, n = 6). (DOCX) [file pone.0192066.s006.docx]

**S2 Table. Bursa to body weight ratio of chickens after vvIBDV inoculation**

| Experiment | Dpi | control | vvIBDV |
| --- | --- | --- | --- |
| Exp. 1 | 3 | 1.3 ± 0.3 | 2.3 ± 0.5* |
|  | 7 | 1.9 ± 0.5 | 0.7 ± 0.2* |
|  | 14 | 1.8 ± 0.5 | 0.6 ± 0.1* |
|  | 21 | 1.5 ± 0.4 | 0.4 ± 0.1* |
|  |  |  |  |
| Exp. 2 | 10 | 1.5 ± 0.3 | 0.5 ± 0.2* |
|  | 14 | 1.5 ± 0.3 | 0.5 ± 0.1* |
|  | 21 | 2.1 ± 0.2 | 0.6 ± 0.2* |

Dpi=days post inoculation; Control=PBS-inoculated control; vvIBDV=vvIBDV-infected group. ^*^letter indicates significant differences between groups at the indicated time point (*P* < 0.05, n=6).
