# Supplementary material for: Infectious bursal disease virus infection leads to changes in the gut associated-lymphoid tissue and the microbiota composition
Source: PLoS One. 2018 Feb 1;13(2):e0192066. doi: 10.1371/journal.pone.0192066 (PMC5794159; doi:10.1371/journal.pone.0192066)
Supplement: S3 Table — dpi = days post inoculation; control = PBS-inoculated control; vvIBDV = vvIBDV-infected group. *indicates significant differences between groups at the indicated time point (P < 0.05, n = 6/group). (DOCX) [file pone.0192066.s007.docx]

**S3 Table.** **Bursa lesion score after vvIBDV-inoculation (Experiment 1 as a representative experiment).**

| Group | Bursa lesion score ± SD  at dpi | | | |
| --- | --- | --- | --- | --- |
|  |  |  |  |  |
|  | 3 | 7 | 14 | 21 |
| Control | 0.0 ± 0.0 (0/6) | 0.0 ± 0.0 (0/6) | 0.0 ± 0.0 (0/6) | 0.0 ± 0.0 (0/6) |
| vvIBDV | 4.0 ± 0.0* (6/6) | 4.0 ± 0.0* (6/6) | 4.0 ± 0.0* (6/6) | 4.0 ± 0.0*^&^ (6/6) |

dpi=days post inoculation; control=PBS-inoculated control; vvIBDV=vvIBDV-infected group. ^*^indicates significant differences between groups at the indicated time point (*P* < 0.05, n=6/group). ^&^Some follicles in bursa sections of vv-IBDV-inoculated birds showed beginning recovery, but depletion of lymphoid cells was still prominent leading to a score of 4 for each bird.
